# Functioning of Women with Migraine Headaches

**DOI:** 10.1155/2014/492350

**Published:** 2014-07-16

**Authors:** Dorota Talarska, Małgorzata Zgorzalewicz-Stachowiak, Michał Michalak, Agrypina Czajkowska, Karolina Hudaś

**Affiliations:** ^1^Department of Preventive Medicine, Faculty of Medical Sciences, Karol Marcinkowski University of Medical Sciences, 11 M. Smoluchowskiego Street, 60-179 Poznań, Poland; ^2^Laboratory of Medical Electrodiagnostics, Department of Health Sciences, Karol Marcinkowski University of Medical Sciences, 49 Przybyszewskiego Street, 60-355 Poznań, Poland; ^3^Department of Computer Science and Statistics, Karol Marcinkowski University of Medical Sciences, 79 Dąbrowskiego Street, 60-529 Poznań, Poland

## Abstract

*Background*. Migraines are one of the most commonly occurring ailments affecting the nervous system. The aim of this research paper was to evaluate the effect migraines have on the everyday functioning of women. *Method*. The study involved women with diagnosed migraine headaches (IHS-2004) undergoing treatment at a neurological clinic. In order to evaluate the influence of headaches on the everyday functioning of women, a MSQ v.2 questionnaire was used, whereas pain severity was assessed on a linear VAS scale. *Results*. Among the clinical factors, the most influential was the frequency of headaches. Headache duration was particularly significant for women below the age of 40. Pain severity cited at 8–10 pts on the VAS significantly disrupted and limited everyday functioning. On the emotional function subscale, the most influential factors were age, education, and the frequency of headaches. *Conclusions*. On account of headache frequency emerging as the most significant influencing factor, it is of the utmost importance to inform patients of the value of taking prophylactic measures. Central to this is the identification of factors that trigger the onset of migraines. This approach would greatly aid the individual in choosing the appropriate treatment, either pharmacological or others.

## 1. Introduction

Migraine headaches are one of the most frequently occurring nervous system ailments. Approximately 12% of the adult population of the United States and Western Europe suffer from them. They occur more often in women, with approximately 15–18% and 6% of the respective populations being noted as sufferers [1–6]. The frequency of migraine headaches, is related to, among other things, changes in female hormones levels [1, 7, 8]. The widespread presence of headaches within society has resulted in the undermining of a systematic neurological approach to treatment. Individuals with headaches most often just seek the help of a GP or undergo self-treatment. Although the medical advice they seek is aimed at reducing the frequency of the occurring pain, they tend to use pharmacotherapy to reduce the headaches rather than administer prophylactic treatment [2, 5, 9, 10].

Migraines, similar to other chronic diseases, influence the psychosocial functioning of patients. They limit the sufferer's abilities both during the onset of pain and between attacks. Acute symptoms limit social relations as well as the capacity to perform professionally and complete household chores [11–14]. In the period between bouts, patients often try to eliminate possible pain triggers; hence they often put limitations on their private and professional lives. In order to assess both the effect of treatment and the influence of headaches on the everyday functioning of patients, research into the quality of their lives was carried out.

Quality of life in relation to health (HRQoL) can be seen as referring to an individual's assessment of their health, functioning (physical and mental), and general well-being [4]. Migraine Specific Quality of Life (MSQ) questionnaires, MIDAS, HIT-6, and SF-36 general health questionnaires are among the most often used questionnaires in relation to adult migraine sufferers. Results from these surveys reveal that the biggest factors affecting quality of life are headache severity and frequency [12, 15–17].

The aim of this research paper was to investigate the influence of migraine headaches on the everyday functioning of women taking into account sociodemographic and clinical factors.

## 2. Methods and Materials

The research involved 125 female outpatients aged 18–60 with a history of migraine headaches. Headache classification was made in line with the diagnostic criteria specified by the International Headache Society (IHS-2004).

### 2.1. Instruments

The Migraine Specific Quality of Life questionnaire (MSQ v.2), complied by Glaxo Welcome and made available by the Medical Outcomes Trust, was used as the main diagnostic tool to assess life quality. MSQ v.2 is an improved version of the first MSQ v.1 questionnaire. It assesses the everyday functioning of women over the most recent four-week period of their lives. It consists of 14 items grouped into three subscales [12, 15, 17–19].

- Role restriction (RR) questions 1–7 determine how the everyday activity of a patient is limited by the disease.
- Role prevention (RP) questions 8–11 analyze to what extend everyday activities are disrupted by the disease or need to be ceased on its account.
- Emotional function (EF) questions 12 –14 determine and evaluate the emotional dimension of the disease such as feelings of frustration or helplessness.

The usefulness of the questionnaire was determined by assessing its accuracy and reliability.

Using multivariate analysis it was concluded that the MSQ questionnaire was valid and accurate. Standard deviations remained on the same level indicating that the tested questionnaire did not require data standardization. Individual items correlated the strongest with the domains to which they were assigned. Using Pearson's *r*, individual correlation coefficients between position and field were tested and revealed similar values. They ranged from 0.71 to 0.85 on the role restriction subscale and from 0.77 to 0.86 on the role prevention subscale and featured slightly higher divergence on the emotional function subscale from 0.63 to 0.86.

Questionnaire reliability was assessed with the help of two internal consistency measurements intraclass correlation (ICC) and *α*-Cronbach coefficient. The questionnaire was to be considered reliable if ICC > 0.5 and *α*-Cronbach were greater than 0.7, though the desired value was >0.9 [20].  Table 1 presents the results of the aforementioned tests carried out on the MSQ v.2.1.

The following tests and procedures were also implemented:

- Visual Analogue Scale (VAS) (ranging from 0–10 points) to assess pain severity;
- a questionnaire to gather demographic and clinical data.

For statistical purposes, the VAS was subdivided into three subgroups of pain severity: weak (1–4 pts), moderate (5–7 pts), and severe (8–10 pts).

Demographical data included age, sex, education, professional activity, marital status, and place of residence.

Clinical data included frequency of migraine attacks, headache duration, severity and pain location, prodromes and symptoms accompanying the migraine, pain-relief both pharmacological and nonpharmacological, and comorbidity. Additionally, questions about lifestyle were included, for example, in reference to the use of stimulants such as coffee, strong tea, alcohol, and cigarettes, as well the application of diets and physical activity and exposure to stress.

### 2.2. Inclusion Criteria

In order to participate in this research study, patients had to be

- undergoing treatment at a neurological clinic,
- suffering from the initial stages of a headache,
- diagnosed with a migraine as defined by IHS guidelines,
- aged over 18,
- consenting to participate in the study.

### 2.3. Statistical Analysis of the Results

Statistical analysis was performed with *U* Mann-Whitney for two groups' comparison and Kruskal-Wallis tests for comparing more than two groups. In case significant differences were found, Dunn's post hoc tests were used to find homogenous groups. Statistical analysis was performed with the use of Statistica PL 10.0 (StatSoft) and StatXact 8.0 (Cytel). All tests were considered significant at *P* < 0.05.

Having analyzed the statistically significant differences between the MSQ v.2.1 domain and the variables, an attempt was made to assess the effect of migraines on the everyday functioning of women taking into account both clinical and sociodemographic factors.

With this in mind, the data was collated and two categories were created for each variable, for example, headache duration: up to and more than 24 hours, headache frequency: chronic and episodic, and age: below and above 40 years.

As headache frequency was the most differentiating factor out of all the factors, an attempt was made to determine whether there would be a direct influence on the functioning of women when headache frequency was considered together with all other variables like age, headache duration, pain severity, education, or marital status. A similar analysis was conducted taking age into account, together with other variables, with the assumption that it would show a statistically significant differentiation in relation to the functioning of women both under and over 40.

## 3. Results

### 3.1. Demographic Factors

The mean age of the studied group of women was 37.02 ± 11.37. The most populous age category was 25–45, which could be seen as a period of high professional and maternal activity (Table 2). The majority of women participating in the study were university (48.8%) or high school educated (31.2%). Ninety (72.0%) respondents were professionally active, 14 (11.2%) were retired, and 9 (7.2%) described themselves as unemployed. Married women or those with a partner numbered 87 and accounted for 69.6%. The majority (89 women, 71.2%) of those surveyed lived in a town.

### 3.2. Clinical Characteristics of the Group

Half of the respondents (51.2%) suffered from headaches 1–3 times a month. In the case of 42 women (33.6%) the pain lasted a whole day, for 34 women (27.2%) the pain lasted for 5 hours, and in the case of 19 (15.2%) the pain lasted over 48 hours. For 36 (28.8%) respondents the pain was located in the frontotemporal region. For 60 (48.0%) women the pain was located in one side of the head, whereas 30 (24.0%) experienced pain in the whole of the head. The majority of respondents (49.6%) noted pain at the 5–7 pts level on the linear VAS scale, reflecting moderate severity. Only 30.4% of respondents reported a migraine with aura. The most frequently cited migraine prodromes were sleepiness (20.0%), irritability (36.0%), scotoma (18.4%), numbness in the limbs (8.0%), dizziness (5.6%), and increased appetite (2.4%).

Symptoms connected with the onset of a headache included nausea/vomiting (80.0%), phonophobia (14.0%), photophobia (22.0%), dizziness (6.4%), speech disorders (2.4%), numbness in the body (9.6%), concentration difficulties (10%), and a lowered mood (10%).

In order to alleviate headache pain, all of the respondents used pharmacotherapy but only 10 (8.0%) administered Sumatriptan. Among additional remedies used, the most common were cold compress (34.4%) and sleep (59.2%). Only 10 (8.0%) of the respondents tried massage, acupressure, or herbal tea. Ten (8.0%) women used Divascan or Ergotamine as a preventive remedy.

Out of all the respondents, 23 (18.4%) reported comorbidity. This included diabetes (4.0%), spine/joint degeneration (4.0%), hypertension (4.0%), nephrolithiasis (2.4%), and allergies (2.4%).

### 3.3. Style and Quality of Life

Eighty (64.0%) women admitted to using stimulants. Thirty-three (26.4%) smoked cigarettes, 60 (48.0%) drank strong coffee, 46 (36.8%) drank strong tea, and 27 (21.6%) drank alcohol, usually wine or beer, more than twice a month. However, they did not drink more than the equivalent of 50 mL of distilled alcohol. Moderate physical activity was done by 41 (32.8%) women. Ninety (72.0%) respondents reported irregular consumption of meals and 38 (30.4%) ate fatty meals and fast food. The main reasons for the latter were excessive workloads or too many classes at university.

Everyday stress was experienced by 45 (36.0%) of the respondents whilst for 30 (24.0%) women stress was something they experienced a few times a week. Only 7 (5.6%) women admitted to having an absence of stress in their lives.

In order to assess the quality of life, the MSQ v.2.1 questionnaire was used. In each domain the following scores were obtained (mean number of pts ± SD): RR 55.93 ± 19.98, RP 66.32 ± 22.14, and EF 62.72 ± 22.77. The maximum number of points that respondents could obtain in each domain was 100.

The quality of life of respondents was analyzed taking into account the influence of headaches on their everyday functioning as well as variables such as age, education, marital status, the frequency of headaches and their duration, and pain severity (Table 2).

Using the Kruskal-Wallis test, statistically significant differences between age and quality of life were only found on the EF (*P* = 0.0206) subscale. The data suggests that younger respondents did not cope as well emotionally due to headaches. Women aged 25–35 experienced the greatest limitations. Women aged over 55 were revealed as the group whose quality of life, according to the EF subscale, was the least affected by headaches. The most varied responses were observed in question 13 that is “*how often do you feel like a burden for others because of your migraine?*” Here negative answers were mainly given by respondents aged 45–55 (*P* = 0.01314).

Statistically significant differences were also observed between education and life quality assessment on the RR (*P* = 0.0136) and EF subscales (*P* = 0.0363) with the use of Kruskal-Wallis test. Headaches were seen to limit the everyday and emotional functioning of women with only primary and high school education the most. Within the highest pain severity subscale, women with high school education reported that headache pains impaired their concentration ability and lowered their energy levels (q.5 *P* = 0.0251). Migraines affected the everyday functioning of women with vocational education the least.

In reference to marital status, respondents who were single reported higher levels of disruption to everyday functioning (RP *P* = 0.0028) due to migraines.

**Figure 1:**
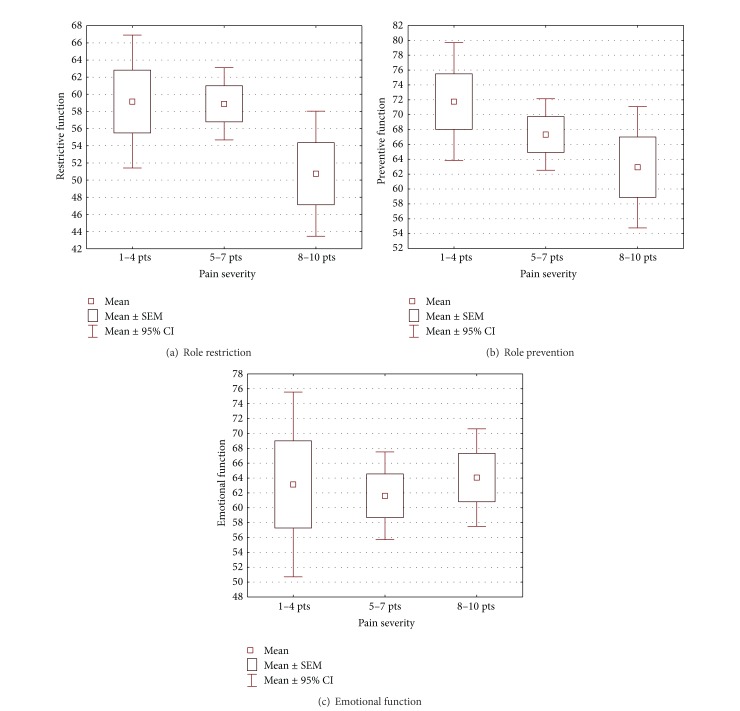
Assessment of life quality versus the severity of pain.

As for the clinical features, headache frequency was most responsible for the greatest significant differences within the life quality assessment (Mann-Whitney: RR *P* = 0.0328, RP *P* = 0.0032, and EF *P* = 0.0089). The more frequent the headaches, the lower the assessment of quality of life on each subscale. Duration and pain severity were found not to have an effect on everyday functioning. Although it was observed that an increase in headache severity increased limitations in performing everyday duties, this dependency was not proven statistically (Figures 1(a), 1(b) and 1(c)). Within the EF subscale, light headaches were seen as being a bigger burden. Due to the significant impact that headaches had on the assessment of life quality, another one of the clinical or demographic factors was added to test whether it would modify respondents' assessment. No statistically important differences were detected for the assessment of each area of life quality when taking into account frequency, severity, age, and education. However, statistically important differences were found in the RP subscale when analyzing the frequency of headaches with marital status (Mann-Whitney *P* = 0.0319). The assessment was lower for women with chronic migraines and living alone. Whereas, in the RR subscale, a significant difference was observed when frequency and duration were analysed together (Mann-Whitney *P* = 0.0171), the assessment in this domain was lower for women with chronic migraines and with headache duration of over 24 hours.

When analyzing the functioning of women in two age groups: below 40 and over 40 (Table 3) it was observed that the functioning of women aged below 40 was dependent on the frequency and the duration of pain. In the group of older women, everyday functioning was influenced by frequency and marital status.

It was also attempted to assess respondents' lifestyle. No statistically significant differences in women's lifestyle were found between those with chronic and episodic headaches.

## 4. Discussion

Migraines are a disease much more commonly found in women than men. In studies investigating large populations, females prevailed among the respondents [1, 2, 5, 10, 13, 19, 21, 22]. Based on these assumptions and findings, for this particular research paper, only women were chosen for the study group. The mean age of the women in this study reflects the most populous female age groups in other studies. In research studies conducted by Rendas-Baum et al. [17], Cole et al. [18], and Martin et al. [12] the mean ages of women were 41.6, 40.7, and 39, respectively. This data coupled with the research of Cevoli et al. [10] which showed that the 30–50 age group accounted for 63.3% of migraine sufferers indicates that the most prevalent participant group was women at their most professionally active in terms of age. For this reason, these studies emphasized the limitations of the disease on the everyday functioning and in particular the professional capacity of women [9, 12–14].

The analysis of mean values from three other MSQ questionnaires conducted by different researchers indicates a medium level of life quality as assessed by their respondents [10, 12, 15]. Within this particular paper, the highest mean score was obtained on the RP subscale (66.32 pts), followed by the EF subscale (62.72) and the lowest on the RR (55.93 pts). The values of the scored means within their separate scales are similar to the results obtained by Cevoli et al. [10].

In that study 953 patients with migraine headaches were assessed and the following results were obtained: RR-50.8 pts, RP-65.4 pts, and EF-62.9 pts. Additionally, studies conducted in the USA, Canada, and Iran among patients with migraines confirmed that the biggest limitations were felt by respondents on the RR scale, while at the same time it was the lowest scored part of the MSQ v.2 questionnaire [17–19]. Among factors that had a significant influence on the everyday functioning of individuals suffering from migraines, Jhingran et al. [15] cited the most common as being frequency, severity, and headache duration. In direct comparison, within this particular paper, the frequency of migraines influenced the assessment of functioning in all MSQ v.2.1 domains, whilst duration only had a significant influence on the RR scale in women aged below 40 and with chronic migraines.

It was revealed, however, that with the increase in the frequency of headaches there was also an increase in the disruption to the everyday functioning of women aged below 40 as well as an increase in limitations in the women over 40. This trend may stem from the fact that the younger women may have had younger children and were in the process of career building so they were better motivated to perform their domestic and professional duties. This assertion is confirmed by research conducted by Bigal et al. [16] in which 50% of students attempted to continue studying despite suffering from a migraine in comparison to only 17% when suffering from a tension headache. Martin et al. [12] in their research revealed a statistically important correlation with migraine frequency and failed to prove the existence of such correlations between pain severity, ability to work, and the domain of life quality. In research conducted by both Lipton et al. [13] and Yavuz et al. [22], 91% and 76%, respectively, of respondents reported limitations in everyday functioning while experiencing a headache. The necessity to lie down was confirmed by approximately 53% of the study participants of Lipton et al. [13] and Bigal et al. [16].

Pain is not the only factor negatively influencing everyday functioning. There are also prodromes and symptoms occurring prior and during a headache attack. Martin et al. [12] stressed the negative impact of vomiting that significantly limited readiness to undertake professional activities. Also, as was found in this paper, accompanying symptoms (vomiting *P* = 0.0344) also limited functioning. Other than physical symptoms, there were also burdensome mental disorders such as irritability or difficulties with concentration that often remained for a period between the attacks [9, 14, 23–25]. Depression is also often a comorbid disorder. Individuals with migraines are 2.2 to 4.0 times more prone to suffer from depression [26]. Pompili et al. [26] showed a two-way relationship between migraines and depression, namely, that the occurrence of one of the disorders increased the risk of the other. Dueland et al. [27] reported an incidence rate of 44% for depression among their respondents. In the research of Innamorati et al. [6], persons with more severe depression perceived themselves as disabled to a greater extend and the symptoms of stagnation were more severe in them.

In this paper, the occurrence of depression was not analyzed. However, 10% of women did report a lowered mood, 48% felt tired for most of the day, and 88% felt they had a lowered concentration ability while experiencing a headache. Among other socioeconomic factors influencing the incidence of migraines, authors draw attention to education, financial situation, and the place of residence, though the collected results vary [5, 8]. In the study on persons with migraine and tension-type headaches (TTH) in Turkey it has been revealed that migraine was present more often in women with lower income and higher education. In Korea migraine pain was more frequent in women living in the rural area whereas income and education level played no role. TTHs were more rare in women living in the rural area with higher education [5–9]. These socioeconomic factors had no influence on results of men in both studies. In our own research women with vocational education reported less limitations caused by the migraine.

The burden of a headache makes patients look for methods to relieve it. Most of the respondents in the study used the treatment designed to stop the migraine pain, most often taking NSAIDs. Triptans were used by less than 10% of the group. Additionally, cold compresses and sleep were applied. In the study by Cevoli et al. [10] 66.6% of respondents took NSAIDs and approximately 17% Triptans. Supplementary pain killers were taken by 1/3 of respondents and 26.4% took over-the-counter drugs. However, Triptans were used by 35.8% of respondents in the study by Malik et al. [2], and only 26.6% took NSAIDs. One-third of the group expressed dissatisfaction with the effects of this drug.

Only a small group of women apply prophylactic treatment. In this research study, this figure was 8% of the study group, whilst in research conducted by Ertas et al. [5] and Cevoli et al. [10] it was about 5%. Prophylactic treatment is used most often by people with chronic migraines. Research conducted by Bigal et al. [16] revealed that 33.3% of the group was taking preventive drugs. The importance of preventive treatment for improving the quality of life has been well explained by Smith et al. [28]. Migraines also have an impact on the functioning of a family. Studies conducted in Canada among mothers with migraine history indicated a negative influence of the mother's disease on the functioning of children [24]. The occurrence of the ailment triggered emotional tension in the family. Women often felt anger and depression. They were rather closed, unwilling to express their feelings, and had lowered spontaneity. Additionally, studies conducted in Brazil by Bigal et al. [16, 29] show that migraine headaches in mothers have negative influences on the functioning of children and increase their headache frequency.

Within this research study, other family members were not included. However, respondents did express feelings of being a burden for others and described frequently experiencing low moods.

## 5. Conclusions

On account of headache frequency emerging as the most significant influencing factor, it is of the utmost importance to inform patients of the value of taking prophylactic measures. Central to this is the identification of factors that trigger the onset of migraines. This approach would greatly aid the individual in choosing the appropriate treatment, either pharmacological or others.

## Figures and Tables

**Table 1:** Intraclass correlation coefficient and *α*-Cronbach for each domain of the MSQ v.2.1 questionnaire.

MSQ v.2.1	ICC	*α*-Cronbach	Correlation between positions
Restrictive function	0.60	0.91	0.61
Preventive function	0.62	0.87	0.63
Emotional function	0.50	0.75	0.51
Total MSQ v2	**0.50**	**0.93**	**0.52**

**Table 2:** Differences between MSQ assessment and demographic and clinical variables.

Demographic and clinical factors	*N* = 125 (%)	MSQ		
		RR mean score	RP mean score	EF mean score
Age				
to 25	16 (12.8%)	53.7	61.6	62.1^a^
25–35	49 (39.2%)	56.8	59.3	62.4^a^
35–45	29 (23.2%)	56.1	57.9	63.4^a^
45–55	25 (20.0%)	52.9	51.8	51.7^a^
over 55	6 (4.8%)	50.0	64.1	83.3^b^
Kruskal-Wallis test (*H* = 4)		1.95	3.33	9.49
Significance level		0.0745	0.5045	**0.0206**
Education				
Primary	3 (2.4%)	52.4^a,b^	56.6	44.4^a^
Vocational	22 (17.6%)	64.2^b^	58.2	63.9^b^
High school	39 (31.2%)	51.1^a^	56.8	62.1^b^
University	61 (48.8%)	54.6^a,b^	58.8	61.1^b^
Kruskal-Wallis test (*H* = 3)		10.68	0.52	8.53
Significance level		**0.0136**	0.9136	**0.0363**
Marital status				
In a relationship	87 (69.6%)	55.9	59.1	60.9
Single	38 (30.4%)	53.2	55.6	62.6
*U* Mann-Whitney test		1384.00	1096.50	5720.50
Significance level		0.1489	**0.0028**	0.1968

Pain frequency				
Chronic	*N* = 39	50.7	58.3	56.1
Episodic	*N* = 86	58.3	69.9	65.7
*U* Mann-Whitney test		1276.5	1126.0	1188.5
Significance level		*P* = 0.0328	*P* = 0.0032	*P* = 0.0089
Pain severity VAS				
1–4	*N* = 17 (13.6)	59.2	71.7	63.1
5–7	*N* = 62 (49.6)	58.9	67.3	61.6
8–10	*N* = 46 (36.8)	50.7	62.9	64.1
Kruskal-Wallis test (*H* = 2)		13.3	6.3	6.17
Significance level		*P* = 0.1252	*P* = 0.6777	*P* = 0.8613
Duration				
To 5 hours	*N* = 34	55.5	60.1	62.7
Whole day	*N* = 42	53.7	54.4	59.7
24–48 hours	*N* = 30	56.2	57.5	60.7
Over 48 hours	*N* = 19	55.7	63.2	64.6
Kruskal-Wallis test (*H* = 3)		3.36	1.24	0.71
Significance level		*P* = 0.3388	*P* = 0.7444	*P* = 0.8714

^a,b^Groups marked with the same letters do not statistically significantly differ.

**Table 3:** Differences in the MSQ assessment taking into account age and selected demographic and clinical data.

Selected criteria		Age to 40			Over 40		
		RR mean score	RP mean score	EF mean score	RR mean score	RP mean score	EF mean score
Pain severity							
1–4	*N* = 17	66.3	70.0	60.0	56.2	72.5	64.4
5–7	*N* = 62	57.6	66.2	61.5	63.3	71.1	61.9
8–10	*N* = 46	50.2	63.3	65.2	51.6	62.4	62.4
Kruskal-Wallis test (*H* = 2)		3.18	0.05	0.44	2.29	0.71	0.27
Significance level		*P* = 0.2042	*P* = 0.9753	*P* = 0.8031	*P* = 0.3184	*P* = 0.7003	*P* = 0.8753
Frequency							
Chronic	*N* = 39	51.8	58.0	56.5	48.6	59.2	55.0
Episodic	*N* = 86	57.6	69.3	65.8	59.3	70.9	65.7
*U* Mann-Whitney test		588.00	474.00	503.00	131.50	138.50	143.0
Significance level		*P* = 0.1955	*P* = 0.0138	*P* = 0.0297	*P* = 0.0581	*P* = 0.1288	*P* = 0.0320
Duration							
To 24 hours		58.03	68.2	65.0	56.0	68.2	65.3
Over 24 hours		51.4	60.7	58.6	57.0	67.3	59.7
*U* Mann-Whitney test		487.00	591.00	613.50	247.50	224.50	230.50
Significance level		*P* = 0.0115	*P* = 0.1368	*P* = 0.2056	*P* = 0.9635	*P* = 0.5669	*P* = 0.6627
Education							
Primary	3 (2.4%)	48.6	60.0	53.3	71.4	82.5	70.0
Vocational	22 (17.6%)	60.7	63.7	57.2	59.1	66.5	55.3
High school	39 (31.2%)	51.7	62.5	59.4	56.9	71.3	63.5
University	61 (48.8%)	56.6	67.8	66.2	52.8	63.9	65.5
Kruskal-Wallis test (*H* = 3)		2.95	1.66	3.44	1.75	1.34	1.20
Significance level		*P* = 0.3997	*P* = 0.6466	*P* = 0.3287	*P* = 0.6261	*P* = 0.7201	*P* = 0.7531
Marital status						70.0	
In a relationship		56.3	66.7	63.1	59.8	73.2	66.7
Single		54.6	63.5	62.0	41.1	42.5	45.0
*U* Mann-Whitney test		719.00	613.00	722.50	76.00	39.00	85.00
Significance level		*P* = 0.7613	*P* = 0.1730	*P* = 0.7868	*P* = 0.0335	*P* = 0.0012	*P* = 0.0622
